# A Data-Driven Analysis of Work-Related Accidents in the Brazilian Mining Sector (2019–2022)

**DOI:** 10.3390/ijerph22060939

**Published:** 2025-06-14

**Authors:** João Oliveira, Anna Luiza Marques Ayres da Silva

**Affiliations:** Mining and Petroleum Engineering Department, Polytechnic School, University of São Paulo, São Paulo 05508-030, Brazil; joaog53009@gmail.com

**Keywords:** workplace safety, mining, occupational health, data analysis, risk perception, storytelling, Brumadinho disaster, occupational accidents

## Abstract

This study applied data analysis techniques to analyze work-related accidents in Brazil’s mining sector from 2019 onward, identifying key risks and patterns. Using public datasets from governmental sources, it categorized accidents by the type of injury, causal agents, and affected body parts. The methodology employed included data cleaning, processing, and the development of interactive visualizations using advanced analytical tools, such as Python and Power BI, to facilitate data interpretation. Among the most significant events, the Brumadinho tailings dam collapse in 2019 emerged as a major outlier, substantially affecting multiple aspects of the analysis. This single incident accounted for 71.7% of all work-related fatalities recorded during the four-year period under study, highlighting its disproportionate impact on the dataset. This study also examined the main causes and consequences of mining accidents and facilitated the creation of victim profiles based on gender and age group, incorporating psychological theories regarding risk perception. It was concluded that, although the mining sector represents a small fraction of all work-related accidents in Brazil, the proportion of accidents relative to the number of workers in the sector is substantial, highlighting the need for stricter occupational safety management. The results can guide regulations and help companies and institutions to create safer, more sustainable mining policies. The methodology proved to be highly suitable, indicating its potential for application in safety analysis across other sectors.

## 1. Introduction

Data analysis has become indispensable across various sectors of the economy, driven by the exponential growth in the capacity to collect, store, and process information. Mining is no exception to this trend; it is now an area where data usage has profoundly transformed the management of operations and processes. Many mining companies employ process intelligence tools, data collected through instrumentation are increasingly integrated with the Internet of Things, and software has become essential for numerical simulations and machinery studies [1,2].

However, in the context of occupational safety, despite the increasing adoption of these technologies and methodologies worldwide, their use remains limited in many countries, such as Brazil. International studies have demonstrated the potential of such approaches for identifying accident patterns and root causes. Altındiş and Bayram [3] applied data mining algorithms such as Apriori and SVM to accident records from underground coal mining in Türkiye, identifying key contributing factors such as worker experience, time of day, and location, and achieving high predictive accuracy in their models. Similarly, Sanmiquel et al. [4] analyzed more than 56,000 accidents in Spanish mining (2005–2015) using the Weka data-mining tool and extracted association rules that highlighted physical exertion as the leading immediate cause in underground accidents, with significant links to worker age and experience.

Both in Brazil and internationally, various organizations collect data on mining-related accidents and work safety. In Brazil, for instance, the Instituto Nacional do Seguro Social (INSS, National Social Security Institute), through the Comunicação de Acidentes de Trabalho (CAT, Communication of Work Accidents), publishes data, and the SmartLab initiative generates visualizations [5]. Internationally, institutions such as the International Council on Mining and Metals (ICMM), the U.S. Bureau of Labor Statistics, and the National Institute for Occupational Safety and Health (NIOSH) also gather such data. Although Brazil’s systems play an essential role in monitoring occupational accidents, comparison with global best practices reveals opportunities for improvement, particularly regarding data standardization, integration, and interoperability. In many cases, the available data remain partially fragmented and lack integration, hindering a comprehensive and accurate analysis of the actual occupational accident scenario in a given country. On the few occasions when data are analyzed, the focus is on macro-level aspects, with minimal detail.

Furthermore, mining has historically been regarded as a high-risk activity, tainted by severe accidents and environmental disasters such as the Brumadinho and Mariana dam collapses, that affect not only local communities but also public perception of the entire industry. The recurrence of accidents in both small- and large-scale mines perpetuates the stigma that the sector is dangerous and environmentally unsustainable. These blemishes on the industry’s image affect companies’ social license to operate, as well as their relationships with investors and service providers, ultimately impacting the viability of operations [6,7].

Therefore, this study proposes the application of a data analysis methodology to understand the main risks in the Brazilian mineral sector, using information from public agencies and various research sources. The distinguishing feature of this research compared to previous studies lies in the integration of tools such as Python and Power BI for processing large volumes of data from Brazilian public databases, as well as in the customized categorization of variables, aligned with the Regulatory Standards currently in force in the country. These elements ensure greater adherence to the local regulatory context, enhance the analytical capacity of the methodology employed, and make the study particularly relevant for scenarios characterized by low data standardization and a lack of integrated analyses. Although the main focus is the Brazilian mineral sector, the suggested approach can be adapted and applied in other countries, contributing to global understanding of mining safety challenges. The main objective is to analyze and interpret data on mining accidents and fatalities, generating graphical materials to facilitate the identification of patterns, trends, and correlations. Additionally, it ensures that preventive measures are based on accurate and representative information.

In this way, this study contributes to the adoption of more effective preventive actions and can support regulatory agencies and industry managers in formulating standards and guidelines to reduce risks and improve working conditions in the mining sector.

## 2. Materials and Methods

**Figure 1:**
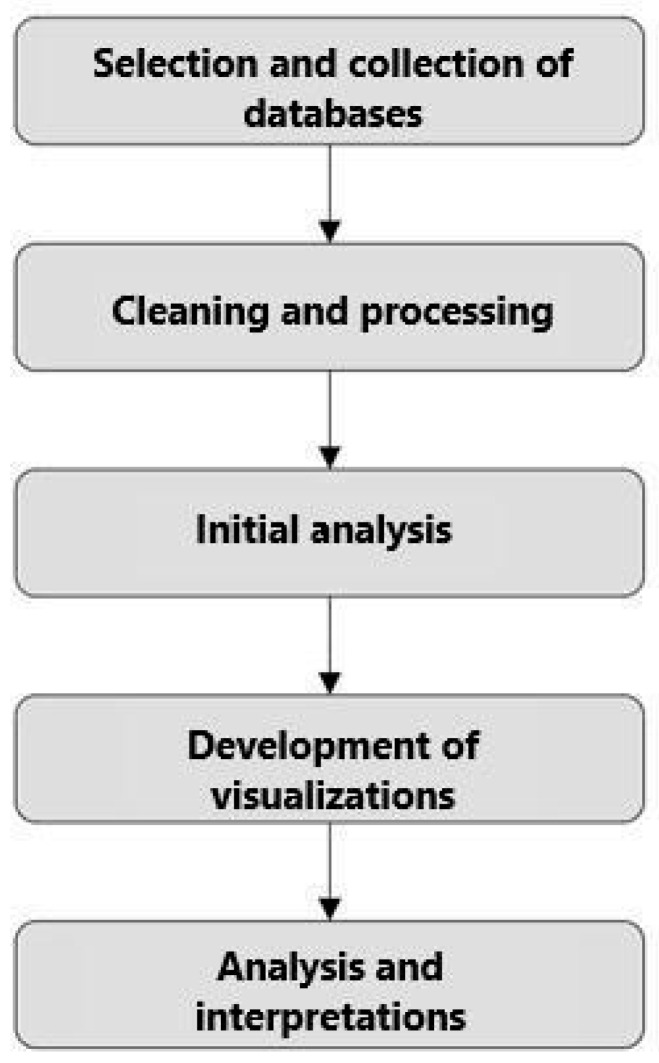
Flowchart of methodology.

The methodology of this study involved collecting data on occupational accidents in the country from reports, dashboards (visual panels containing information, metrics, and indicators), and CAT databases. To achieve this, a standardized sequence for the use and manipulation of the information was employed, as shown in Figure 1.

1. Selection and database collection: This step involved identifying and selecting which sources were going to be used. The reliability of the sources and the relevance of the data for analysis were verified, including the dates of the information, how they were categorized, and finally, extracting the data.
2. Data cleaning and processing: After the selection, the data were prepared for analysis. This process included data cleaning, aimed at correcting inconsistencies, handling missing values, and removing duplicates, ensuring quality and reliability for the analysis.
3. Initial analysis: To preliminarily understand the data, initial handling was conducted to better comprehend how each dataset behaved individually.
4. Development of visualizations: This stage involved creating various charts and tables, using Python graphing libraries such as Matplotlib, as well as other software, like Power BI. The objective was to validate and choose the best way to visualize the data among several possible options.
5. Analysis and interpretations: The generated visualizations were analyzed to extract significant insights regarding accidents and fatalities in the mining sector. This analysis focused on identifying risk areas, critical periods, and common types of accidents, providing a solid foundation for future discussions and in-depth studies.

### 2.1. Database Selection

The data source chosen for the analysis was the open data portal of the Work Accident Communication (CAT) organized by the National Social Security Institute (INSS) (2025) [8]. The CAT is the official document used in Brazil, primarily issued by employers, to record work-related accidents and occupational diseases. This site provides data from July 2018 to May 2023. The data from 2019 to 2022 were selected as the focus of analysis since these years offer complete periods for evaluation.

### 2.2. Data Cleaning and Processing

The data cleaning and processing phase involved a mix of manual handling through Excel, Python scripting, and operations in Power Query (the data transformation tab in Power BI). It is important to highlight that in data science, *data cleaning*, unlike *data filtering*, refers to the removal of invalid or inconsistent records to improve the quality of the dataset without applying filters. Overall, the selected data were relatively well structured and consistent.

Initially, to improve the organization of the files, the quarterly or monthly data from each year were manually consolidated into a single file per year. What were originally 20 “.csv” (comma-separated values) files became 4 “.xlsx” (Excel) files with standardized column names. One of the quarters in 2020 contained several thousands of incomplete accident entries, where all the characteristics were marked as “unclassified.” It was understood that this was an error in the file creation, given the excessively large number of rows with this pattern, so they were excluded.

Next, a Python script was employed, primarily using the Pandas library, which efficiently handles file reading, table operations, and data analysis. This script consolidated the “.xlsx” files into a single dataframe (a Pandas object that can be understood as a table in Python) to detect and address column inconsistencies. The final step of the script exported this dataframe as a “csv” file.

The first inconsistency found was the presence of text in the “*Data Acidente*” (Accident Date) column, preventing the transformation of the column into a datetime object, but only eight rows presented this error, so they were removed. The second inconsistency was 2495 null dates (00/00/0000) in the “*Data de Nascimento*” (Date of Birth) column. These anomalies could disrupt the analysis or cause problems in Power BI, but they represented a negligible fraction of the total 1,876,726 rows (approximately 0.13%), so they were removed from the dataset.

The final processing stage took place in Power BI/Query. After loading the unified “.csv” file, the “*Data de Nascimento*” (Date of Birth) column was transformed from a date type with a “dd/mm/yyyy” format into the “*Idade*” (Age) column, represented as a numerical type. Additionally, an “*Faixa Etária*” (Age Group) column was created to group the age values into more comprehensible categories for analysis and visualization. An “*Ordem Faixa Etária*” (Age Group Order) column was also added to ensure that Power BI would sort the age groups by a defined order from youngest to oldest, rather than alphabetically. To ensure transparency and facilitate reproducibility, all source codes developed in this research are provided in Appendix A.

### 2.3. Initial Analysis

During the data cleaning and preprocessing stage, columns such as the employer’s CNPJ and the accident issuance date were excluded, as they were not relevant to the primary focus of the study. The CNPJ (“Cadastro Nacional da Pessoa Jurídica”), which identifies individual companies in Brazil, was removed due to data privacy concerns and because the analysis was conducted at a macro level, rendering company-specific details unnecessarily granular. Similarly, the accident issuance date was excluded given that the actual date of the accident provides more meaningful information for the analysis and was therefore prioritized.

The initial analysis focused on categorizing the characteristics of the accidents at a macro level, as the available level of detail was too granular, which could hinder future analyses. This stage also involved establishing relationships between the fact table and the dimension tables.

It is important to remember that, in data analysis, the fact table serves to store the quantitative data of a business or event, typically referencing dimension tables [9]. In this study, the fact table recorded accident data.

Dimension tables contain descriptive data that provide context to the fact table, such as dates, products, or customers. They are used to avoid redundant information in the fact table [10].

#### 2.3.1. Categorization

The macro-level categorization of mining accident data, as shown in Table 1, Table 2, Table 3 and Table 4, followed specific criteria to group similar items under broad categories. This approach made it easier to analyze and understand the various aspects of accidents (causal agents, affected body parts, nature of the injuries, and employment sectors).

Regarding the categorization of causal agents, the classification criteria provided by the Regulatory Standard NR-1 [11] were applied, which classifies agents as physical, chemical, or biological. However, the categories of exertion and reflex do not fit into any of these three types, so they were complemented by NR-17 [12], classifying them as ergonomic agents. It is important to note that physical agents include (but are not limited to) noise, vibrations, pressure, extreme temperatures, and ionizing and non-ionizing radiation.

It is worth noting that the Regulatory Standards (“Normas Regulamentadoras” (NR)) in Brazil are a set of mandatory guidelines established by the Brazilian Ministry of Labor and Employment to ensure occupational health and safety across various work environments. These standards set minimum requirements for workplace conditions, risk management, and worker protection across different industries. They are aligned with international best practices, drawing on recommendations and guidelines from the International Labour Organization (ILO) to enhance occupational health and safety standards [13]. The categorization of accident-causing agents reflects a pragmatic approach, based on empirical knowledge of the mining context and on functional distinctions widely recognized in the field of occupational safety. Although no strict standardized taxonomy was applied, the separation between categories such as “tools” and “machines,” for example, is justified by clear differences in modes of operation and levels of risk exposure. Manual tools, such as hammers and chisels, involve direct physical effort and contact-related risks, while heavy machinery, such as crushers and hydraulic presses, pose automated mechanical risks, often of greater severity. This classification aimed to balance interpretative clarity with analytical usefulness, as detailed in Table 1, and aligns with industry conventions while supporting the practical goals of the analysis.

This categorization process is essential for identifying patterns and implementing effective preventive measures. Table 1, Table 2 and Table 3 provide the descriptions used for each categorization.

While the factors mentioned above have multiple categories, even after simplification, the analysis of the employer’s sector was binary. Given that this study focused solely and exclusively on mining activities, there were only two categories: “mining” and “non-mining.” Table 4 shows the activities considered as mining.

Spelling errors and incomplete words were intentionally left uncorrected to avoid discrepancies with the databases, where they appear in their original form. Correcting the text would make data verification more difficult, as software considers any difference in characters as distinct words.

In addition to these tables, a Calendar Table was created, covering all days, even those without accidents, from 01/01/2019 to 12/31/2022. These dates were further broken down into columns for year, month (both number and text), quarter, and the start of the month. The “*Começo do Mês*” (Start of the Month) column is extremely important for allowing analysis by month and year without the granularity of individual days and without loss of information. For example, all days in January 2019 have the value 01/01/2019 as the start of the month.

#### 2.3.2. Table Relationship

Data processing is an iterative process of trial and error. As analyses are prepared, new inconsistencies and areas of concern are discovered that need to be addressed. This step is vital to ensure the quality of the columns, so that the interpretations are correct and accurate, and to allow Power BI to function without error notifications.

The data were loaded into Power BI for processing in Power Query. After a simple treatment to remove columns that would not be used in the analysis, the categorization tables (Table 1, Table 2, Table 3 and Table 4) were imported, the Calendar Table was created, and the relationships were established to begin the data analysis. The accident database served as the fact table, and the others as dimension tables.

**Figure 2:**
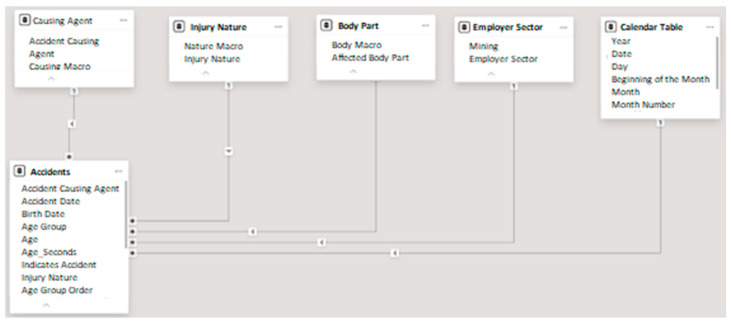
Relationship map in English.

The relationships established between the fact table and the dimension tables were all of the “one-to-many” type. In other words, a single record from one table could be associated with multiple records in the other, while each record in the second table was related to only one in the first. This type of relationship was implemented by using a foreign key in the table with multiple records, referencing the primary key of the table with the unique record. Since the dimension tables originated from the fact table, referential integrity was guaranteed, but it was still verified. Operationally, the relationships were established by dragging the corresponding columns from one table to another. The column names did not have to match, only the content. Figure 2 shows the relationship map of the tables used.

This step enables visual interactions among multiple graphs through a process known as cross-filtering. When a user selects a specific element within a visual, such as a bar, slice, or column, Power BI dynamically filters the data displayed in the other visuals on the same report page. For example, selecting the “Female” slice in a pie chart causes all related visuals to adjust accordingly, displaying only the subset of data relevant to female participants. Consequently, bar and column charts are recalibrated, showing updated values and percentages reflective of the filtered data set. This interactive capability enhances exploratory data analysis by allowing users to focus on specific data segments in a visually intuitive manner [14].

## 3. Results and Discussion

To facilitate the reading and comprehension of this study, the last two stages of the methodology (“Development of visualizations” and “Analysis and interpretations” in Figure 1) were combined for each analysis. The graphs support deeper exploration of the various aspects of mining accidents, such as gender, the main accident-causing agents, key impacts, and temporal and age-related analyses.

### 3.1. Brazilian Mining Sector Overview

One of the simplest analyses that can be conducted is identifying the percentage of accidents attributed currently to the mining industry and comparing it to the total number of people employed in the sector. Only direct employment was considered in this analysis.

**Figure 3:**
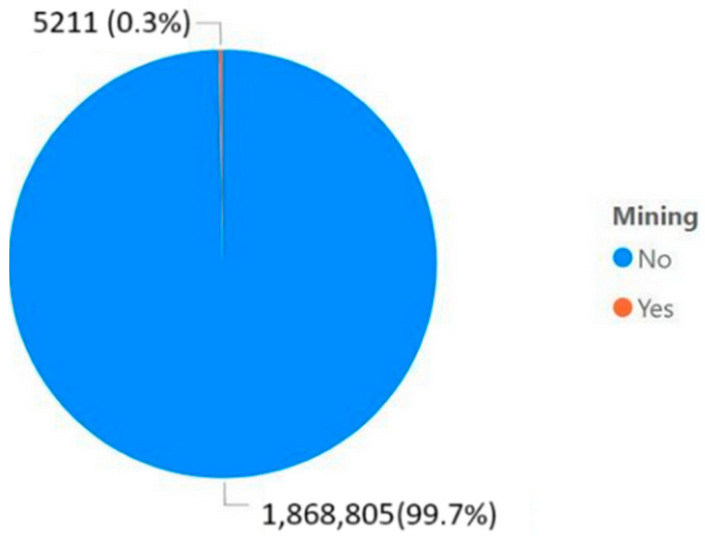
Total accidents in Brazil.

As shown in Figure 3, only 0.28% (5211 out of 1,874,016 total accidents) of the accidents recorded between 2019 and 2022 occurred in the mining sector, which might suggest that the situation is not particularly severe. However, in 2022, the mining industry employed only 0.20% of Brazil’s working population, or 205,000 individuals [15] out of a total of 100.7 million [16]. In other words, the sector’s representation in accidents is proportionally higher than its share of the workforce.

Another measure extracted from the database is that, in 2022, 1902 accidents occurred in the mining sector. Assuming each accident involved a different person, nearly 1% (0.92%) of those directly employed in mining were involved in some form of accident. On the other hand, only 21 of these accidents resulted in fatalities. This figure differs from the data reported in the Safety Performance Report [17], which indicates that only four fatalities occurred in Brazil. This discrepancy arises from the fact that not all mining operations are affiliated with ICMM, a global organization that brings together mining and metals companies and associations to promote responsible and sustainable practices in the industry.

**Figure 4:**
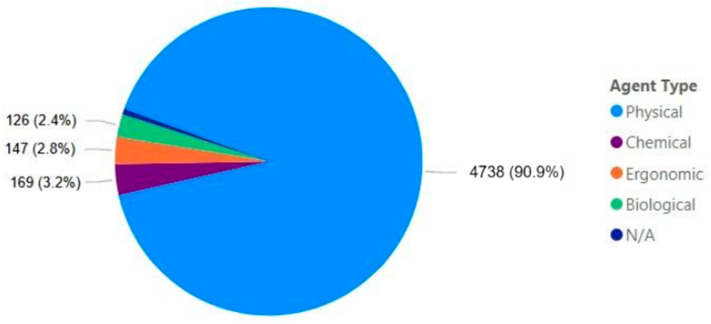
Mining accidents by type of agent.

As shown in Figure 4, of the 5211 accidents, the majority (90.9%) were caused by agents classified as physical. The other three types (chemical, ergonomic, and biological) accounted for roughly the same proportions (3.2%, 2.8%, and 2.4%, respectively), while the remaining accidents were not classified. These types of agents are categorized as NR-01 and NR-17, and it is expected that physical agents dominate, as their definition is relatively broad and encompasses various subtypes. Physical agents are defined in the general provisions as “any form of energy that, by its nature, intensity, and exposure, is capable of causing injury or harm to the worker’s health” [11].

**Figure 5:**
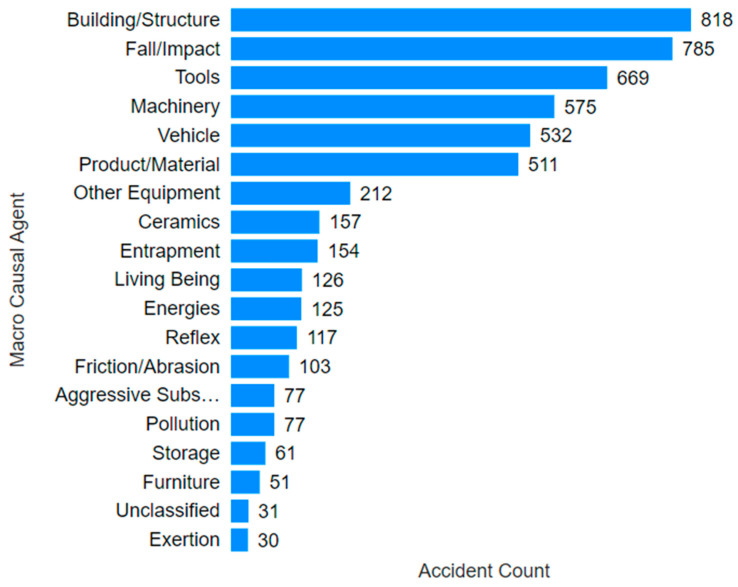
Distribution of mining accidents by causal agent.

Through increasing the level of detail, Figure 5—which focuses on the agents causing mining accidents—highlights the most prominent agents. It is evident that the top six agents accounted for 74.6% of all accidents in the sector, and 14 out of the top 20 agents were classified as physical.

**Figure 6:**
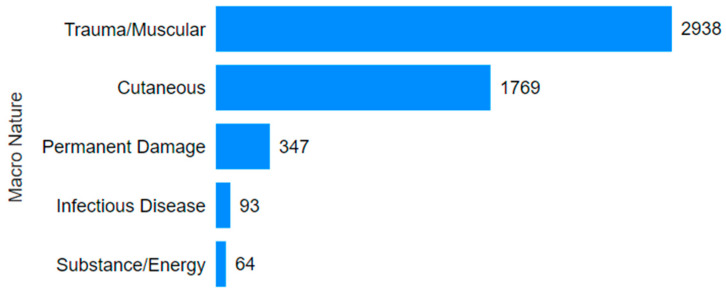
Injury type in the mining sector.

**Figure 7:**
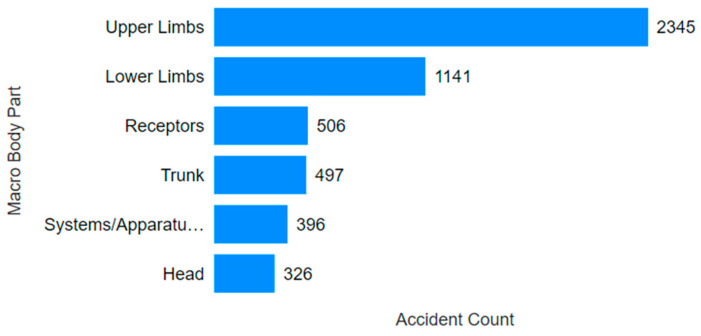
Affected body part in the mining accidents.

Consulting Figure 6 and Figure 7, 90.3% of the accidents involved trauma/muscular and skin damage. Regarding the affected body parts, 66.9% of accidents involved either the upper or lower limbs. The most frequent outcomes included fractures, sprains, strains, dislocations, or cuts, lacerations, abrasions, and burns to the shoulders, arms, hands, legs, feet, and fingers. These accidents are relatively easy to visualize: a fall resulting in a sprain or fracture, a finger being broken due to a tool mishap, or a cut during the maintenance of equipment with sharp edges. Using Power BI’s data segmentation, it was found that in 93 cases (approximately 2.7%) of accidents involving the upper and lower limbs combined, the victims sustained permanent damage (amputation or loss of function). Power BI enables interactive filtering, whereby selecting a specific injury type allows users to visualize how these cases relate to outcomes such as fatalities or serious injuries, including amputations. Through this functionality, it was possible to isolate and examine subsets of more severe incidents.

Another notable finding is the low incidence of infectious diseases, even though the entire COVID-19 pandemic was included in the years analyzed, which spanned from January 2019 to December 2022.

Finally, performing data segmentation again, it was discovered that 152 fatalities were recorded in the sector during this period. Of that number, 109 resulted from the Brumadinho disaster, which will be addressed in the next section.

### 3.2. Brumadinho Disaster

An irreparable catastrophe for those affected and a significant stain on the mining sector’s reputation, the collapse of the Córrego do Feijão Mine dam on 25 January 2019, in Brumadinho, Minas Gerais, was one of the worst tragedies caused by the mining industry. The disaster resulted in 272 deaths in total [18] (including non-employees), psychological trauma for the survivors, and profound environmental impacts on the Paraopeba River basin [19]. This section aims to understand the accident from the perspective of workplace safety and its impact on data analysis. At no point is there an intention to dehumanize the victims by treating them solely as statistics, but rather to comprehend the numbers behind the accident.

**Figure 8:**
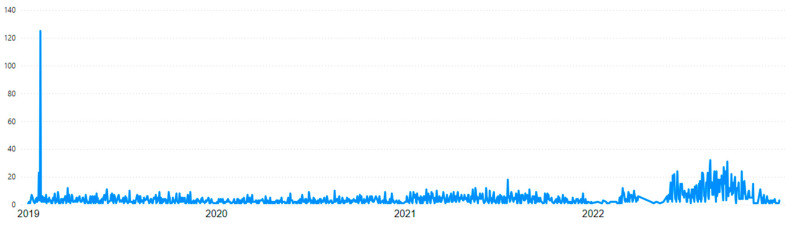
Timeline of mining accidents by date.

The first visual that highlights the event is the timeline of mining accidents in Figure 8, which specifically focuses on accidents by date without grouping them by week or month. The peak at the beginning of the graph stands out as an outlier compared to the rest of the data, significantly altering the proportion of the chart and making the remaining line appear flatter. Although the Brumadinho disaster resulted in 272 fatalities, the dataset employed in this study includes only work-related accidents, thereby excluding non-employee victims from the analysis. Specifically, the data records 117 occupational accidents associated with the event, of which 109 resulted in fatalities.

**Figure 9:**
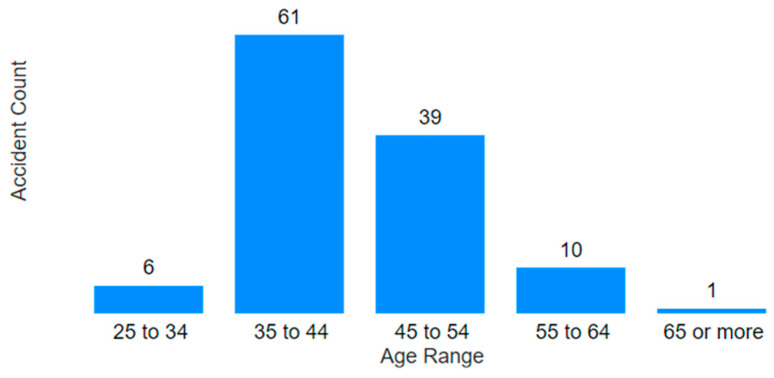
Age range of the victims.

**Figure 10:**
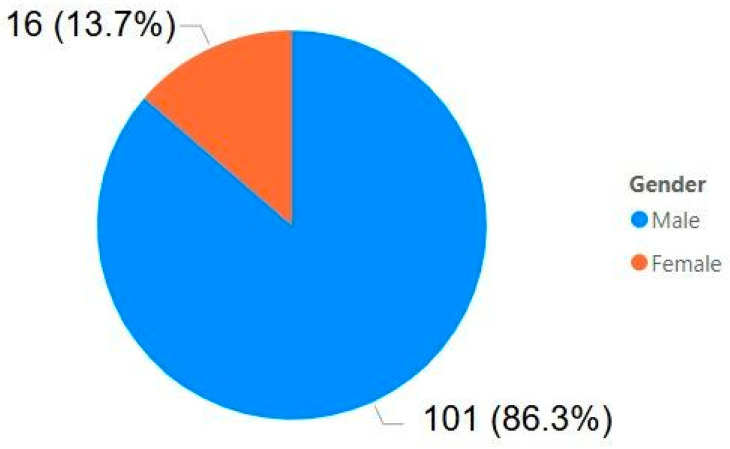
Gender of the victims.

Based on the information from Figure 9 and Figure 10, it is evident that the majority of the affected employees were between the ages of 35 and 54, a group in the middle-to-late stages of their professional careers. Most of the victims were men, which is an expected outcome considering that mining is a predominantly male industry. In total, 117 employees were impacted and, after filtering the data in Power BI, it was found that only 8 men survived, suffering permanent injuries, trauma, and even contracting infections.

This day should not be forgotten from the collective memory, as it was a disaster that claimed more lives in a few hours than the entire sector had in four years. In September 2020, Law 14,066 was enacted, spurred by this event, updating the National Dam Safety Policy (PNSB) to prevent similar catastrophes from occurring in other locations [20,21].

### 3.3. Accidents by Gender

Over the years, the presence of women has been increasing in predominantly male sectors, such as information technology, engineering, and others. Mining is following this trend; globally, women represent between 8% and 17% of the mining workforce [22], with Brazil positioned closer to the upper range, where 15% of the workforce is female [23].

Regarding the analysis of accidents by gender, only data excluding the Brumadinho disaster were considered, specifically entries with dates other than 25/01/2019. This exclusion was motivated by the fact that the event had no correlation or causation related to gender, as it is known to be gender-agnostic. As a result, the number of accidents analyzed was reduced to 5086.

**Figure 11:**
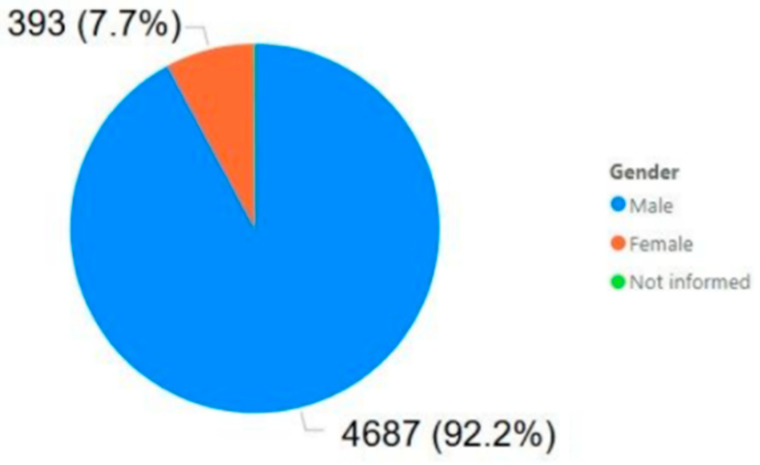
Mining accidents by gender.

Figure 11 shows the quantities and percentages of mining accidents by gender. It was anticipated that the male share would be the majority; however, it is noteworthy that women represent less than 8% of accidents, slightly over half of their overall share in the sector. This suggests that, in mining, the accident rate among women is considerably lower than that of men.

Statistical analysis was conducted to determine whether the proportion of women involved in mining accidents differed significantly from their representation in the workforce. Among the 5086 recorded accidents, 393 involved female workers, representing an observed rate ($\hat{p}$) of 7.7%. A two-tailed test for proportions was applied, comparing this observed rate to the expected rate (p_0_) of 15%, based on women’s representation in the mining workforce. The test yielded a z-score of approximately −14.50 (Equations (1) and (2)) and a *p*-value of less than 0.0001 (*p* ≈ 1.23 × 10^−47^) [24], indicating a highly significant difference. These findings provide solid statistical evidence that women in mining experience accidents at a significantly lower rate than expected based on their workforce representation, reinforcing the conclusion that their accident rate is substantially lower than that of their male counterparts.

(1) $$z=\left(\hat{p}-p_{0}\right)/\surd \left[p_{0}\;\left(1-p_{0}\right)/n\right]\;z$$

z = (0.077 − 0.15)/√[0.15 (1 − 0.15)/5086] ≈ −14.50(2)

where

$\hat{p}$ = sample proportion (proportion of female accident cases) = 0.077;

p_0_ = expected population proportion (female participation in the sector) = 0.15;

n = sample size (total number of accidents analyzed) = 5086.

**Figure 12:**
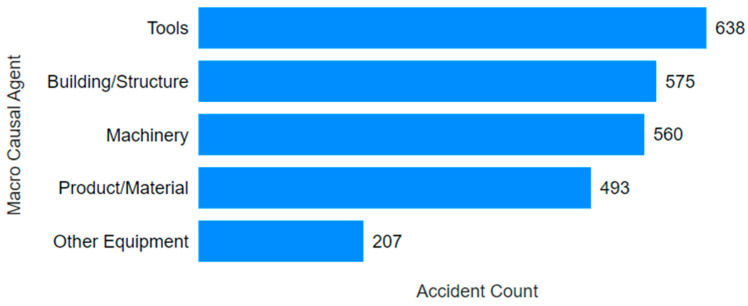
Main causal agents for males.

**Figure 13:**
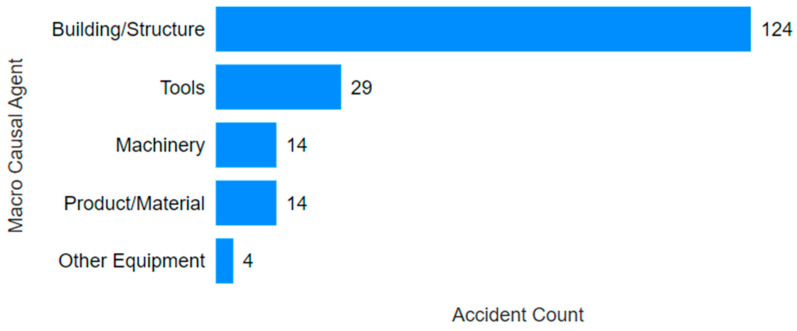
Main causal agents for females.

Another aspect highlighted by the visuals (Figure 12 and Figure 13) is the difference in the main causal agents for each gender, with accident frequency as the criterion of importance. Although both groups exhibited the same top five causal agents, the frequency differed. For males, the accidents were more evenly distributed across the top four types (tools, building/structure, machinery, and product/material), with a sharp drop from the fourth to the fifth. In contrast, for females, most accidents were concentrated in the building/structure category, with a nearly 77% lower rate of tool-related accidents. Machinery- and product/material-related accidents were about half as frequent as tool-related ones.

However, caution must be applied when interpreting these figures. As highlighted by Heimann et al. (2023) [25], the mining sector remains marked by horizontal and vertical gender segregation: women are disproportionately represented in administrative and support functions, while men are dominant in high-risk operational roles, particularly underground. This occupational stratification significantly affects exposure to hazards and must be acknowledged as a potential confounding factor in accident data. In addition, the masculinized culture of mining, often emphasizing physical toughness and risk acceptance, may influence accident underreporting by men and shape the types of tasks women are assigned, further skewing comparative statistics. Another factor that may distort gender-based comparisons is the difference in recovery duration: women tend to take longer sick leaves following accidents, which can reduce their frequency in datasets based on lost workdays [26].

### 3.4. Age Analysis

Delving further into the profile of the individuals involved in accidents, seven distinct age groups were defined, as shown in Table 5. The “Age Group” column was added to Power BI as a conditional column based on the “Age” column, and an additional column was created to order these age groups in ascending order. This step was necessary because Power BI treats values as text and would otherwise sort them in alphanumeric order.

Similar to the gender analysis, the cases from the Brumadinho disaster were filtered out for the age-based analysis, as there was no correlation between age and the event.

**Figure 14:**
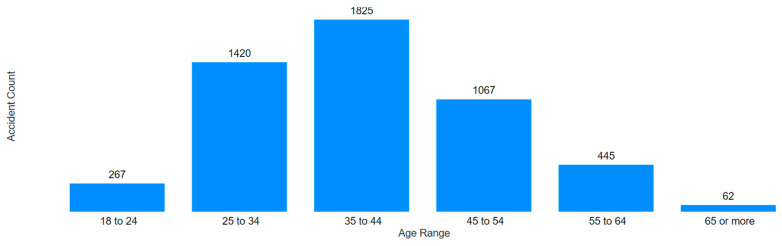
Accidents by age range.

The first aspect highlighted in Figure 14 is the absence of accidents involving underage workers (up to 17 years old). This suggests a heightened concern for young apprentices in the sector and/or indicates that they may be more attentive to safety norms and protocols. Tian et al., in a study on mining-related injuries in Chinese coal mines, also observed a lower incidence of injuries among younger workers (<20 years), which potentially reflects the limited inclusion of workers under formal contracts within this age range. This was mainly attributed to their reduced involvement in critical operations, greater supervision, and a more cautious approach due to a lack of overconfidence, which could lead to risky behaviors [27].

Secondly, the age groups with the highest number of occurrences were noted to be those between 25 and 54 years, collectively comprising 84.3% of all accidents. A rise in accidents was observed from the 25–34 to the 35–44 age group, followed by a 42.2% decline from the 35–44 to the 45–54 group.

Finally, groups with fewer accidents included ages 18–24, 55–64, and 65 or older. It is possible that fewer professionals are active in these age groups, either due to retirement among the older groups or lack of qualifications among the youngest.

It could be inferred that the occurrence of accidents is related to the number of professionals within each age group. However, updated demographic data on mining professionals are only available in the country for coal mining in the Brazilian states of Santa Catarina and Rio Grande do Sul, which was deemed insufficient for generalizing across the rest of the country [28]. Therefore, psychological and cognitive explanations were preferred for analyzing the accident distribution by age, focusing on the risk perception in the workplace. Risk perception is an entirely subjective factor; each person analyzes and interprets a given risk differently, potentially underestimating or overestimating probabilities and consequences.

The study “Adult Age Differences in Risk Perception and Risk Taking” by Nolte and Hanoch [29] analyzed different risk perceptions by age group, drawing on established theories to explain the correlation. Risk sensitivity theory posits that needs, benefits, and costs are key motivational factors in how risks are approached. When linked to age, younger adults who have not yet achieved sufficient status or financial comfort may be more inclined to take greater risks to reach their goals, whereas older adults who have satisfied these needs are less likely to do so. Additionally, socioemotional selectivity theory suggests that older individuals tend to prioritize more positive outcomes, aiming to avoid scenarios with potential losses.

Regardless, both younger and older individuals rely on the availability heuristic [30]; therefore, the cognitive mechanism is the same across ages. The major differentiator in decision making is the individual’s prior experience; those who have witnessed or experienced minor accidents with minimal consequences may underestimate new risks, whereas those who have seen serious accidents with significant impacts may have their perceptions deeply altered, making them more cautious in future situations [31].

Applying this psychological and cognitive foundation to mining in Brazil, we might assume that age groups at both ends of the spectrum are more cautious: the younger (up to 24 years old) due to mining’s hazardous image and fear of accidents, and the older (above 55 years old) due to their experience with accidents and cognitive factors. The intermediate age groups (25–54 years) may reflect a mix of factors: unmet ambitions and needs, years of job experience without witnessing severe accidents, and overconfidence, among others. This culminates in a lower risk perception among individuals in this age group, leading them to take more risks during their workday.

These findings are supported by studies conducted with Swedish and Serbian miners, which indicate a higher incidence of accidents among middle-aged workers, while younger and older workers tend to be less involved in incidents, either due to lower exposure or greater caution acquired through experience [32,33]. However, Ghosh et al. [34] observed that workers over the age of 45 showed a higher likelihood of being involved in accidents, particularly when factors such as work-related stress, job dissatisfaction, and emotional instability were present. This suggests that older age alone does not guarantee greater risk perception and may even be associated with specific vulnerabilities [34].

It is important to emphasize that this age-based analysis, grounded in psychological and cognitive factors, does not aim to attribute blame for accidents to individuals but rather to explore potential explanations on an individual level. The primary responsibility for safety rests with the employer, who must foster a safety culture beginning with upper management, setting goals, and encouraging safe practices across all organizational levels.

### 3.5. Temporal Analysis

In Section 3.2, regarding the Brumadinho Disaster, Figure 8 presents a timeline of accidents by date, covering every day from 01/01/2019 to 12/31/2022 without omission. However, such a granular level of detail hinders a macro-level analysis, especially given the presence of a highly significant peak that distorts the graph’s proportions. Therefore, grouping time periods is the optimal solution to enable temporal data analysis, allowing for adjustments across different levels of detail.

The three levels of analysis to be considered are month/year, month, and year. The “month/year” level aggregates all days within a specific month of a given year, such as January 2019, February 2019, and so on. This grouping facilitates the construction of an accident timeline without daily granularity, making it easier to observe monthly trends over the years. The “month” and “year” levels, respectively, provide an analysis of data across all months and all years, as their names suggest. Alternative groupings, such as week or quarter, were deemed unsuitable as they represent excessively short or overly extended periods, which could compromise the consistency and relevance of the analyses.

**Figure 15:**
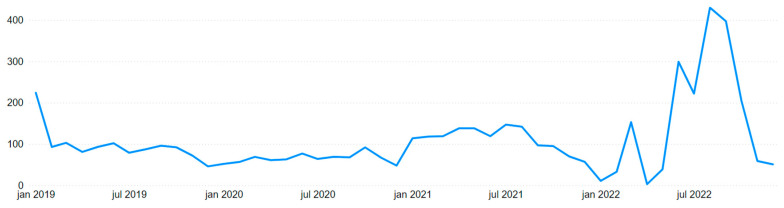
Timeline of mining accidents by month/year.

**Figure 16:**
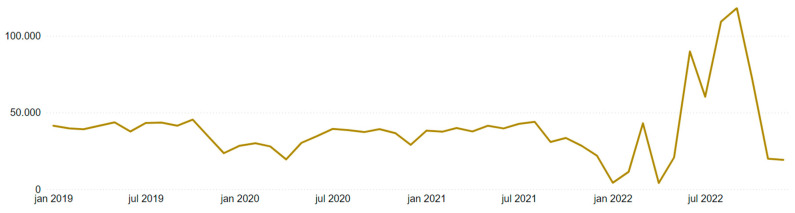
Timeline of Brazilian accidents by year/month.

Figure 15 and Figure 16 present two graphs with similar formats but different scales. Figure 15 is limited to mining accidents only, while Figure 16 encompasses all employer sectors.

In mining, there was a peak in January 2019 due to previously mentioned events, followed by a relatively stable trend until December 2021, with a sharp decline in January 2022. Notably, 2022 appeared turbulent for mining, displaying high variability: January and April showed very few occurrences, while values were extremely high from June to October.

Except for the January 2019 peak, Brazil’s overall timeline followed the same pattern from January 2019 through December 2021, after which the high variability in 2022 began. The fact that the abnormal behavior in 2022 was mirrored both with and without the mining filter suggests that these data may be compromised in some way. Therefore, this behavior was further investigated.

**Figure 17:**
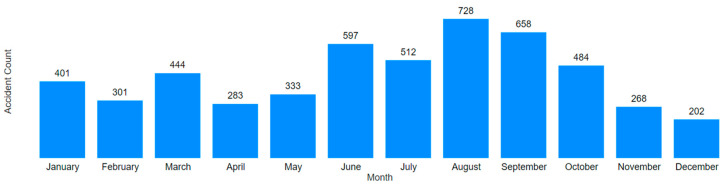
Monthly distribution of mining accidents.

**Figure 18:**
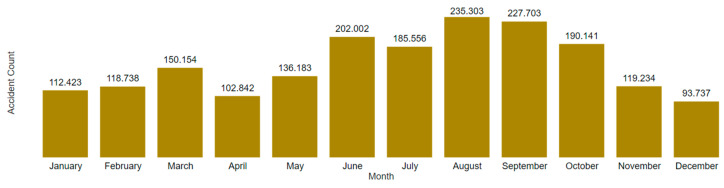
Monthly distribution of Brazilian accidents.

In analyzing the monthly accident trends in both mining and Brazil as a whole, Figure 17 and Figure 18 again show similar temporal patterns regardless of the filter applied. Utilizing Power BI’s visual interaction feature, it is possible to filter column charts via timelines, and vice versa, to extract further insights from the data.

For the months in 2022 with unusually high values (June to October), accident rates fluctuated around 50% of all accidents in those months across all years. For instance, in mining, August 2022 accounted for more than half of the total accidents recorded in August across all years, while for Brazil, this phenomenon occurred in September. Furthermore, in a year-over-year comparison, these months showed up to a fourfold increase compared to the previous year (regardless of the filter applied). April 2022, representing a trough, was also proportionally extreme. Nationally, April reached only 11% of the previous year’s count, and in mining, it was merely 2%.

**Figure 19:**
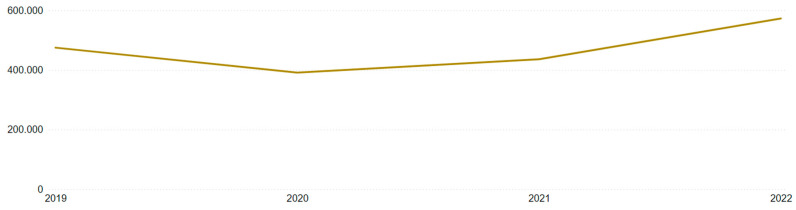
Annual distribution of Brazilian accidents.

**Figure 20:**
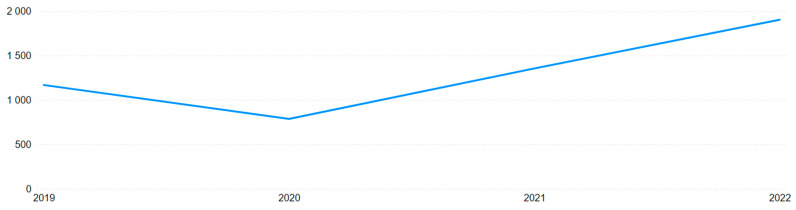
Annual distribution of mining accidents.

Incorporating the annual accident distribution from Table 6 into the analysis revealed a 17.5% decrease from 2019 to 2020, followed by a 31.2% increase from 2021 to 2022 across Brazil. This “bathtub” curve, visible in Figure 19 and Figure 20, reflects the impact of the COVID-19 pandemic and the restrictions it imposed between 2020 and 2021, with a rebound in 2022. However, it still does not fully explain the previously noted monthly anomalous behavior.

In addition to the data analysis, potential explanations for the temporal anomalies were investigated. While one possible interpretation is that these inconsistencies stem from errors in data reporting or processing within the CAT system, such as delayed or bulk submissions, no official sources were found to substantiate this claim. As no external documentation confirms a systematic issue during this period, the explanation remains a hypothesis and should be treated as a tentative interpretation rather than a definitive conclusion. Consequently, the irregular distribution of accident records across certain months does not appear to reflect a genuine seasonal pattern.

## 4. Conclusions

This study demonstrated that, although the mining sector represents a small fraction of total employment in Brazil, it exhibits a disproportionately high rate of occupational accidents. The predominance of physical causal agents, particularly involving tools and machinery, and the concentration of injuries in the limbs through trauma and skin lesions reflect the operational hazards intrinsic to the sector. The Brumadinho disaster significantly skewed fatality statistics, underscoring the critical impact of large-scale failures on sectoral safety profiles.

Stratified analyses revealed important demographic patterns: men accounted for the majority of accidents due to occupational segregation, while workers aged 25–54 were the most affected, possibly due to reduced risk perception. Possible temporal inconsistencies in 2022 raise concerns about the data reliability but are not confirmed.

To address these findings, the study recommends (i) enhancing the accuracy and timeliness of reporting systems, (ii) prioritizing physical hazard mitigation strategies, (iii) implementing age- and role-sensitive safety training, and (iv) promoting gender equity in operational roles. Additionally, it calls for greater regulatory oversight of informal mining activities in order to make the data reflect all the workers, not only a part of them.

The methodological framework employed—integrating data cleaning, categorization, and visualization—proved effective and scalable, offering valuable support for evidence-based safety policies within and beyond the mining industry.

Future studies will aim to build on this foundation by investigating specific causal relationships across different mining sub-sectors, such as open-pit versus underground operations. This initiative may contribute to the development of more accurate and effective diagnoses, further strengthening prevention strategies and risk management in occupational safety within the mining industry.

## Figures and Tables

**Table 1:** Macro categorization of accident causal agents.

Causal Agent Category	Agent Type	Description
Entrapment	Physical	Entrapment of limbs or the entire body between objects or resulting from collapses.
Storage	Physical	Includes containers (barrels, boxes, crates, etc.), equipment (tanks, silos, barns, etc.), and storage facilities.
Friction/Abrasion	Physical	Abrasions from various sources (handling of equipment, vibrations, compressions, etc.).
Ceramics	Physical	Objects made of ceramic materials such as dishes, bricks, glassware, and others.
Fuels/Oils/Flammables	Chemical	Alcohol, gasoline, paraffin, and other oils.
Building/Structure	Physical	Accidents caused by structures where workers may pass through or work on, including scaffolding, ladders, bridges, viaducts, excavations, etc.
Energies	Physical	Includes accidents caused by exposure to different forms of energy, such as electrical, thermal, or mechanical, including electric shocks or exposure to extreme temperatures.
Exertion	Ergonomic	Accidents in this category are related to injuries caused by excessive physical exertion, such as lifting, pushing, or pulling heavy objects.
Tools	Physical	Includes incidents involving the use of manual or mechanical tools.
Machinery	Physical	Accidents related to large machinery or other heavy equipment used in various operations.
Furniture	Physical	Includes accidents associated with furniture and accessories.
Other Equipment	Physical	A collection of various types of equipment that do not fit into other categories, including electrical and lighting equipment.
Pollution	Chemical	Refers to accidents caused by exposure to environmental pollutants, such as noise, air, and water pollution.
Product/Material	Physical	Includes incidents related to the handling or exposure to various final/intermediate products or raw materials.
Fall/Impact	Physical	Accidents resulting from falls or impacts, including people falling or colliding with or from objects.
Reflex	Ergonomic	Categorizes accidents that occur as reactions to involuntary or voluntary movements that result in injury.
Unclassified	N/A	Not classified.
Living Being	Biological	Includes accidents involving animals or other living organisms, such as bites, attacks, or parasitism.
Aggressive Substance	Chemical	Includes incidents of exposure to substances that can cause direct harm, such as acids, alkalis, or toxic compounds.
Vehicle	Physical	Accidents related to the use or handling of vehicles of any type, motorized or not.

**Table 2:** Macro categorization of affected body parts.

Affected Body Parts Category	Description
Head	Includes all injuries to the head, covering the skull, brain, and multiple parts of the head.
Upper Limbs	Includes injuries to the upper limbs, including shoulders, arms, forearms, elbows, hands, and fingers.
Lower Limbs	Covers all injuries to the lower limbs, including thighs, knees, legs, ankles, and toes.
Trunk	Includes injuries to the abdomen, back, chest, hips, and pelvis, considering both the external surface and internal organs.
Systems/Apparatuses	Includes injuries affecting entire bodily systems, such as the respiratory, digestive, circulatory, urinary, musculoskeletal, and nervous systems.
Receptors	Includes injuries to sensory organs, such as eyes, ears, nose, and mouth.

**Table 3:** Macro categorization of injury type.

Nature of the Injury Category	Description
Trauma/Muscular	Includes fractures, bruises, crush injuries, strains, sprains, dislocations, inflammations of joints, tendons, or muscles, and immediate injuries.
Infectious Disease	Covers contagious or infectious diseases and unspecified general morbid conditions.
Cutaneous	Groups cuts, lacerations, abrasions, thermal burns or scalds, dermatoses, and chemical burns.
Permanent Damage	Includes multiple injuries, loss or impairment of senses, such as hearing or vision, amputations, and conditions resulting in permanent loss of function or sensation.
Substance/Energy	Includes electric shocks, poisonings, immediate or delayed effects of radiation, pneumoconiosis, and other effects of harmful substances or energies.
Breathlessness	This category is dedicated to asphyxia, strangulation, drowning, and other conditions that impair adequate breathing.

**Table 4:** Mining sector employer.

Mining Sector Employer
Extraction of Minerals
Extraction of Ores
Extraction of Precious Metal Ores
Extraction of Non-Metallic Minerals Not Specified
Manufacture of Non-Metallic Mineral Products
Extraction of Aluminum Ores
Extraction of Minerals for Fertilizer Production
Extraction of Iron Ore
Extraction of Non-Ferrous Metallic Minerals
Support Activities for Mineral Extraction
Extraction of Coal
Extraction of Manganese Ore
Extraction of Radioactive Minerals
Extraction of Tin Ore

**Table 5:** Age groups.

Age Group (Years)	Age Group Order
Up to 17	1
18 to 24	2
25 to 34	3
35 to 44	4
45 to 54	5
55 to 64	6
65 or more	7

**Table 6:** Annual distribution of accidents.

Year	Brazil	Mining
2019	474,597	1168
2020	391,107	787
2021	435,939	1354
2022	572,373	1902
Total	1,874,016	5211

## Data Availability

The original contributions presented in this study are included in the article. Further inquiries can be directed to the corresponding author.

## Appendix A

The following link provides access to the folder containing the Power BI, Python, and Excel files developed in this study: https://drive.google.com/file/d/15IMEdrebL11WwlI65lsjdOqslG14PiBA/view (accessed on 6 June 2025).
